# Process factors explaining the ineffectiveness of a multidisciplinary fall prevention programme: A process evaluation

**DOI:** 10.1186/1471-2458-8-332

**Published:** 2008-09-24

**Authors:** Michel HC Bleijlevens, Marike RC Hendriks, Jolanda CM van Haastregt, Erik van Rossum, Gertrudis IJM Kempen, Joseph PM Diederiks, Harry FJM Crebolder, Jacques ThM van Eijk

**Affiliations:** 1 Department of Social Medicine, Faculty of Health, Medicine and Life Sciences, Maastricht University, PO box 616, 6200 MD Maastricht, The Netherlands; 2 Department of Health Organisation Economics and Policy, Faculty of Health, Medicine and Life Sciences, Maastricht University, PO box 616, 6200 MD Maastricht, The Netherlands; 3 Department of Clinical Epidemiology and Medical Technology Assessment, University Hospital Maastricht, PO Box 5800 6202, AZ Maastricht, The Netherlands; 4 Department of Health Care and Nursing Science, Faculty of Health, Medicine and Life Sciences, Maastricht University, PO box 616, 6200 MD Maastricht, The Netherlands; 5 Faculty of Health and Technology, Professional University Zuyd, Nieuw Eyckholt 300, 6400 AN Heerlen, The Netherlands; 6 Department of Healthcare studies, Faculty of Health, Medicine and Life Sciences, Maastricht University, PO box 616, 6200 MD Maastricht, The Netherlands; 7 Department of General Practice, Faculty of Health, Medicine and Life Sciences, Maastricht University, PO box 616, 6200 MD Maastricht, The Netherlands; 8 School for Public Health and Primary Care (Caphri), Faculty of Health, Medicine and Life Sciences, Maastricht University, PO box 616, 6200 MD Maastricht, The Netherlands

## Abstract

**Background:**

Falls are a major health threat to older community-living people, and initiatives to prevent falls should be a public health priority. We evaluated a Dutch version of a successful British fall prevention programme. Results of this Dutch study showed no effects on falls or daily functioning. In parallel to the effect evaluation, we carried out a detailed process evaluation to assess the feasibility of our multidisciplinary fall prevention programme. The present study reports on the results of this process evaluation.

**Methods:**

Our fall prevention programme comprised a medical and occupational-therapy assessment, resulting in recommendations and/or referrals to other services if indicated. We used self-administered questionnaires, structured telephone interviews, structured recording forms, structured face-to-face interviews and a plenary group discussion to collect data from participants allocated to the intervention group (n = 166) and from all practitioners who performed the assessments (n = 8). The following outcomes were assessed: the extent to which the multidisciplinary fall prevention programme was performed according to protocol, the nature of the recommendations and referrals provided to the participants, participants' self-reported compliance and participants' and practitioners' opinions about the programme.

**Results:**

Both participants and practitioners judged the programme to be feasible. The programme was largely performed according to protocol. The number of referrals and recommendations ensuing from the medical assessment was relatively small. Participants' self-reported compliance as regards contacting their GP to be informed of the recommendations and/or referrals was low to moderate. However, self-reported compliance with such referrals and recommendations was reasonable to good. A large majority of participants reported they had benefited from the programme.

**Conclusion:**

The results of the present study show that the programme was feasible for both practitioners and participants. Main factors that seem to be responsible for the lack of effectiveness are the relatively low number of referrals and recommendations ensuing from the medical assessments and participants' low compliance as regards contacting their GP about the results of the medical assessment. We do not recommend implementing the programme in its present form in regular care.

**Trial registration:**

ISRCTN64716113

## Background

Falls are a major health threat to older people living in the community, and initiatives to prevent these falls should be a public health priority. Approximately one third of community-dwelling people aged 65 and over fall at least once a year [1-6]. About one fifth of all falls result in an injury that requires medical attention, and about one tenth lead to serious physical consequences, such as fractures, joint dislocations and lacerations [6-9]. In addition, falls can have considerable psychosocial consequences, like fear of falling, depression and social isolation [10-12]. Together, these physical and psychosocial consequences are responsible for reduced physical activity [11,13], early admission to hospital or nursing home [2,14], increased mortality and morbidity [14,15] and loss of autonomy [2,10].

Close and colleagues developed a multidisciplinary fall prevention programme aimed at community-dwelling people aged 65 years and over who had visited the accident and emergency (A&E) department because of a fall [16,17]. Although this programme showed promising effects in this British setting, this is no guarantee for its effectiveness in other healthcare settings. We therefore developed a Dutch version of this successful programme and tested its effect on falls and daily functioning by means of a randomized controlled trial [17]. The results of this trial showed that the programme did not have any effect on falls or daily functioning [18]. In parallel to this randomized controlled trial, we carried out a detailed process evaluation primarily aimed at assessing the feasibility of our multidisciplinary programme. The second aim of this process evaluation was to identify factors which might explain the lack of effectiveness of our programme. This paper presents the results of this process evaluation. We translated the two aims of our evaluation into the following four specific research questions:

(a) To what extent was the fall prevention programme performed according to protocol?

(b) What was the nature of the recommendations and referrals made to the participants?

(c) What was the participants' self-reported compliance?

(d) What are the participants' and practitioners' opinions about the programme?

## Methods

### Fall prevention programme

The fall prevention programme consisted of a medical and occupational-therapy assessment, followed by recommendations or further referral if indicated. The medical assessment consisted of examinations performed by a geriatrician, a geriatric nurse and a rehabilitation physician at the hospital [17]. The assessment included a comprehensive general examination and a detailed assessment of vision, sense of hearing, locomotor apparatus, feet and footwear, peripheral nervous system, mobility, balance, anthropometry, cognition, affect, blood test if indicated and medication use. On completion of the medical assessment, the geriatrician evaluated the results and sent a written summary to the participant's general practitioner (GP). This letter included recommendations and/or referrals to relevant services, if necessary. The participants were advised to contact their GP to be informed of the results of the medical assessment and the recommendations and/or referrals to other services ensuing from it.

The occupational-therapy assessment was performed by an occupational therapist at the participant's home and comprised a functional and environmental assessment [17]. On completion of this assessment, recommendations with regard to behavioural change, functional needs and safety within the home environment were immediately given to the patient. Recommendations and referrals concerning technical aids and adaptations or additional support to be provided by social and community services were implemented in accordance with the procedures prevailing in regular care. The participants received a letter with the recommendations and/or referrals, by way of reminder. A copy was sent to the participants' GPs, to inform them of the results of the assessment.

### Usual care

The participants who were allocated to the control group of the randomized controlled trial and for that reason did not underwent the fall prevention programme, received usual care. During the trial, no standard approach to fall risk assessment was available for fallers presenting to the A&E department and being discharged home. In usual care in the Netherlands, medical risks and other risk factors for falls, such as environmental hazards in the home and patients' risk behavior, are not systematically registered and addressed by hospital physicians, medical specialists or general practitioners. Moreover, when people present to the A&E department with the consequences of an injurious fall, in general no systematic attention is being paid to the specific consequences of that fall for daily functioning of individual patients in their unique situation.

### Study population

The study population of this process evaluation can be divided into two groups:

1. All 166 participants allocated to the intervention group (referred to below as participants).

2. The medical and paramedical practitioners who performed the medical and occupational-therapy assessments (one geriatrician, three geriatric nurses, two rehabilitation physicians and two occupational therapists) (referred to below as practitioners) [17].

### Data collection

Table 1 shows the aspects of the intervention process that were assessed and the methods used. Data were collected from participants by means of self-administered questionnaires and structured interviews by telephone. Independent assistants asked the participants to fill out a questionnaire immediately after the medical assessment in order to assess their opinion about this assessment. For practical reasons and to avoid social desirable answers, the participants did not receive a questionnaire from the occupational therapist immediately after the occupational-therapy assessment. In order to assess the participants' opinion about the occupational-therapy assessment, detailed questions about this assessment were embedded in the structured telephone interviews which took place about six months after the recommendations and referrals had been sent to the GPs. These telephone interviews also comprised questions assessing participants' compliance with the referrals and recommendations and their overall opinion about the programme.

**Table 1:** Outcome measures and measurement instruments of the process evaluation

	Events in chronological order →					
	R	Q	FI	L	T	PD
Performance of programme according to protocol						
▪ Deviations from protocol	X		X			X
▪ Timing and duration of the assessments	X	X	X			X
Nature of recommendations and referrals from assessments				X		
Participants' compliance with referrals and recommendations						
▪ Self-reported compliance with contacting GP					X	
▪ Self-reported compliance with referrals and recommendations resulting from the medical assessment					X	
▪ Self-reported compliance with recommendations resulting from the occupational-therapy assessment					X	
Opinion about the programme						
▪ Benefit and satisfaction experienced by the participants		X			X	
▪ Practicability of the recording forms			X			
▪ Acceptability of the programme to participants			X			
▪ Recommendations (for implementation)			X			X

R = Structured recording forms for the practitioners regarding the medical and occupational-therapy assessments; Q = Self-administered questionnaires for all participants who underwent the medical assessment; FI = Structured individual face-to-face interviews with the practitioners; L = Letters written by the geriatrician and occupational therapists to GPs, listing recommendations and/or referrals; T = Structured interviews by telephone with the participants who underwent the medical and/or the occupational-therapy assessment, about 6 months after the recommendations ensuing from the assessment(s) had been sent to the GP; PD = Plenary group discussion with the practitioners and the research team.

We used structured recording forms, structured face-to-face interviews and a plenary group discussion to collect data from the practitioners regarding the performance according to protocol, the nature of the recommendations and referrals, the compliance of the participants with the referrals and recommendations, and their opinion about the programme. The recording forms were filled out by the practitioners during or immediately after the assessments. The structured face-to-face interviews with the practitioners were scheduled immediately after all participants had undergone the assessments, and the plenary group discussion with the practitioners and the research team was carried out six months after all participants had undergone the assessments.

### Data analysis

Quantitative data (e.g. duration of the assessments, perceived benefit) were analyzed by means of descriptive statistics. Qualitative data (i.e. answers to open questions in the self-administered questionnaires, individual interviews and the plenary group discussion) were classified into categories, based on the content of the answers given.

### Ethical considerations

The Medical Ethics Committee of Maastricht University and the University Hospital Maastricht approved this process evaluation, being a part of the randomized controlled trial [17].

## Results

### Attendance and response rate

**Figure 1:**
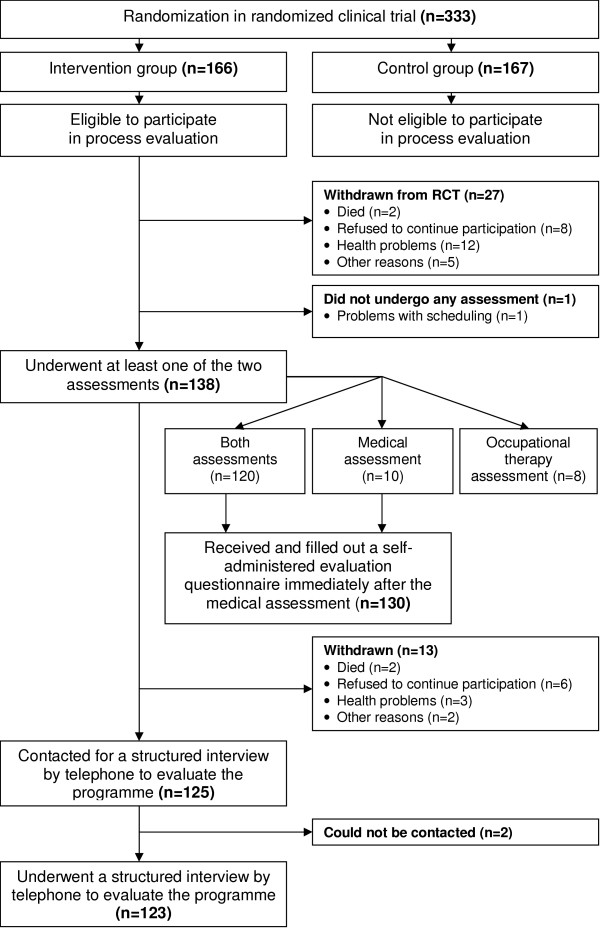
Flow chart of participants.

The flow of participants through the process evaluation is shown in figure 1. Of the 166 persons allocated to the intervention group, 28 (17%) did not undergo any assessment because they withdrew from the study before the start of the assessments (n = 27) or had a problem with scheduling the assessments (n = 1). A total of 138 participants underwent at least one of the two assessments: 120 underwent both assessments, ten only the medical assessment and eight only the occupational-therapy assessment. Reasons for undergoing only one assessment were personal circumstances (n = 14) and withdrawal from the study before the occupational-therapy assessment was scheduled (n = 4). None of these reasons were related to the programme. All 130 participants who underwent the medical assessment received a self-administered evaluation questionnaire immediately after the medical assessment. The response to this questionnaire was 100%. Of the 138 participants who underwent at least one assessment, thirteen withdrew from the study after completing the medical and/or occupational-therapy assessment. The remaining 125 participants were contacted for a structured interview by phone, about six months after the results of the assessments had been sent to the GPs. Two persons could not be contacted, resulting in a response of 98%. Of these 123 participants, 116 had undergone the medical assessment and 117 had undergone the occupational-therapy assessment.

The practitioners filled in recording forms during the assessments for all 130 participants who underwent the medical assessment and for all 128 participants who underwent the occupational-therapy assessment. All but one practitioner (an occupational therapist) took part in the structured face-to-face interviews immediately after the implementation period of the programme. In addition, the practitioners, except one geriatric nurse and one rehabilitation physician, participated in the plenary group discussion six months after the last assessments.

### Performance of programme according to protocol

#### Protocol deviations

The recording forms filled in by the practitioners showed that 97% of the protocol items were carried out according to protocol. Analyzing the recording forms revealed only one minor protocol deviation. During the medical assessment blood pressure was not measured in the erect position (in stead of measuring both sitting and in erect position) in 28 of the 130 participants (22%). The information obtained from the forms was in agreement with the information gathered during the face-to-face interviews and the plenary group discussion.

#### Duration of the assessments, time between baseline measurement and sending the letters with recommendations

The geriatrician, the geriatric nurses and the rehabilitation physicians reported that it took 60 to 90 minutes to perform the medical assessment. The mean amount of time the geriatrician spent processing the referrals and recommendations to the GPs was estimated to be 15 minutes. The mean duration of each occupational-therapy assessment was 55 minutes and the mean time spent on processing a recording form was 21 minutes. The reported time needed for the medical and occupational-therapy assessments was in agreement with the protocol. The period between baseline measurement and sending letters to the GPs with recommendations was on average 3.5 months.

### Nature of the recommendations and/or referrals

#### Referrals and recommendations resulting from the assessments

Table 2 shows the nature of the referrals and recommendations ensuing from the medical and occupational-therapy assessments. The referrals and recommendations made by the geriatrician comprised referrals to other specialists or therapists and recommendations concerning measures such as change of medication and orthopaedic footwear. The recommendations made by the occupational therapists can be subdivided into four categories: (1) adaptations to the home environment (e.g. installing hand rails, shower chair, raised toilet); (2) behavioural change (e.g. adapting speed of working, using antiskid mats, removing loose rugs, using hand rails); (3) health services (e.g. intake for assistive living, intake for a home for the elderly, GP consultation); and (4) assistive devices (e.g. walking device, lift chair).

**Table 2:** Referrals and recommendations resulting from the medical and occupational-therapy assessments

	Number of R/R resulting from assessments
*Referrals from Medical assessments (n = 130)*	
Cardiologist	8
Osteoporosis examination	8
Orthopaedic shoemaker	25
Orthopaedic instrument maker	1
Physiotherapist	4
Other referrals	4

**Total**	**50**
	
*Recommendations from medical assessments (n = 130)*	
Adjust medication	7
Adjust footwear	3
Further examination	8
Vitamin B supplementation	2
Other recommendations	5

**Total**	**25**
	
*Recommendations from occupational-therapy assessments (n = 128)*	
Adaptations to the home environment	134
Behavioural change	301
Health services	6
Assistive devices	16

**Total**	**457**

* R/R = referral/recommendation

As reported by the geriatrician, the medical assessments resulted in 50 referrals and 25 recommendations for the 130 participants, which is on average 0.58 referrals/recommendations per participant. Forty-three percent of the participants (n = 56) received at least one referral or recommendation, and 57% (n = 74) received no referral or recommendation.

As reported by the occupational therapists, 128 participants received a total of 457 recommendations (3.57 per participant) during the occupational-therapy assessments. For 91% of the participants (n = 117), the occupational-therapy assessment resulted in at least one referral or recommendation. For 9% (n = 11), it did not result in any referral or recommendation.

Overall, of the 138 participants who underwent at least one of the two assessments, 123 participants (89%) received at least one recommendation or referral.

### Participants' compliance

#### Contact with GP

Of the 123 persons interviewed by telephone, 7 had not undergone a medical assessment and could therefore not answer the question whether they had contacted their GP. Of the remaining 116 participants, about half (n = 61) had contacted their GP to ask for the outcomes of the medical assessment, 45% (n = 52) had not contacted their GP and 3 persons (2%) did not answer this question. Reasons for not contacting the GP were: forgotten (n = 28); not being aware of the possibility to contact the GP (n = 13); still intending to contact the GP (n = 6); not considering it necessary to contact the GP (n = 4) and death (n = 1).

#### Self-reported compliance with recommendations and referrals

**Figure 2:**
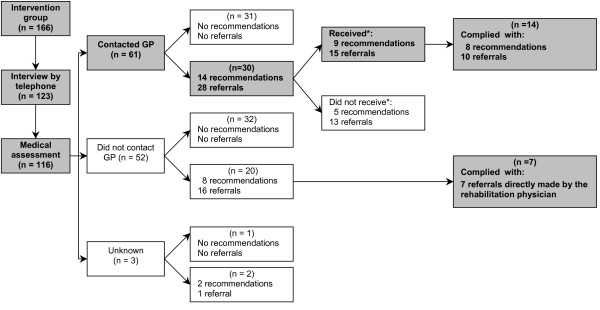
**Net implementation of the referrals and recommendations ensuing from the medical assessment**. *) no numbers of participants are presented due to overlap between categories (some participants did only receive part of the referrals and/or recommendations).

Figure 2 reports on the net implementation of the referrals and recommendations ensuing from the medical assessments. For 30 of the participants who contacted their GP (n = 61), the medical assessment resulted in 28 referrals and 14 recommendations. After the implementation period of the programme, 14 participants reported that 8 recommendations and 10 referrals had actually reached them through the GP and had been implemented. For 20 participants who did not contact their GP, the medical assessment resulted in 16 referrals and 8 recommendations. Because these participants did not contact their GPs, these referrals and recommendations did not reach the participants. However, 7 participants complied with the referral to an orthopaedic shoemaker even though none of them had contacted their GP, because the referral was made directly by the rehabilitation physician during the medical assessment.

**Figure 3:**
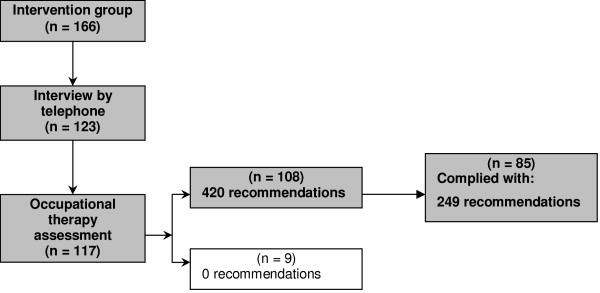
Net implementation of the recommendations ensuing from the occupational-therapy assessment.

Figure 3 reports on the net implementation of the recommendations ensuing from the occupational-therapy assessments. A total of 108 participants received 420 recommendations. At the end the implementation period of the programme, 95 of these 108 participants reported that they had received and complied with 249 recommendations.

Table 3 shows the results on the nature of the referrals and recommendations ensuing from the medical assessment for those participants who called their GP and the participants' self-reported compliance. As it is not possible to comply with referrals and/or recommendation one did not receive, we calculated the compliance for those participants who actually called their GP and reported that they received referrals and/or recommendations from their GP. Overall, the participants who called their GP and received referrals and/or recommendations complied with 18 out of 24 referrals and recommendations, a compliance of 75%.

**Table 3:** Referrals and recommendations resulting from the medical assessment for those participants who called their GP, and participant's self-reported compliance

	R/R* resulting from medical assessment	R/R* received from GP	Self-reported compliance
*Referrals*			
Cardiologist	5	3	3
Osteoporosis examination	7	2	1
Orthopaedic shoemaker	11	9	6
Orthopaedic instrument maker	1	0	-
Physiotherapist	2	1	0
Other referrals	2	0	-

**Total referrals**	**28**	**15**	**10**
			
*Recommendations*			
Adjust medication	3	3	3
Adjust footwear	3	2	1
Further examination	3	0	-
Vitamin B supplementation	1	1	1
Other recommendations	4	3	3

**Total recommendations**	**14**	**9**	**8**
			
**Total referrals and recommendations from medical assessment**	**42**	**24**	**18 (75%)**

* R/R = referral/recommendation

Table 4 shows the results on the nature of the recommendations ensuing from the occupational-therapy assessment and the participants' self-reported compliance. Overall, the participants reported having complied with 59% of the recommendations they had received from the occupational-therapy assessment.

**Table 4:** Recommendations ensuing from the occupational-therapy assessment and self-reported compliance with these recommendations

	R* made to participant	Self-reported compliance
*Recommendations*		
Adaptations to the home environment	124	68 (55%)
Behavioural change	279	174 (62%)
Health services	6	3 (50%)
Assistive devices	11	4 (36%)

**Total**	**420**	**249 (59%)**

*R = recommendation

### Participants' and practitioners' opinions about the programme

#### Participants' opinions about the programme

During the telephone interviews, a majority of the participants reported that they had benefited from the assessments. This percentage was 82% for the medical assessments and 80% for the occupational-therapy assessments. Overall, 84% of the participants reported that they had perceived at least some benefit from the programme as a whole. Besides the perceived benefit, the participants were also asked whether they were satisfied with the medical and occupational-therapy assessments. Almost all participants were satisfied, viz. 97% and 99% for the medical and occupational-therapy assessments, respectively (ranging from somewhat satisfied to very satisfied).

### Practitioners' opinions

The practitioners were asked to give their opinion about whether the participants had benefited from the programme. They judged that most participants had benefited, particularly those who received recommendations for footwear, adaptations to the home environment, or assistive devices. In addition, they thought that in most cases the participants were satisfied with the programme. Although the practitioners were optimistic about the programme benefits, they reported that in their opinion a considerable proportion of the participants, i.e. those with only minor health problems, should not have been included in the trial. The practitioners considered it unlikely that these persons would benefit much from the programme.

The practitioners judged the programme to be feasible and considered all aspects included in the assessments relevant. They considered the recording forms to be easy to work with, although some aspects could be improved, such as the structure and layout of the forms. They also mentioned two aspects that should be added to the programme protocol: a pre-printed list of medications that increase the risk of falling and an instrument to assess fear of falling.

The practitioners were also positive about their own role in the programme. However, they mentioned that there should be more interdisciplinary consultation and communication between the practitioners to agree on referrals and recommendations. Moreover, both assessments should be more closely tailored to the needs of individual patients and more assessments and training should be done in the home environment. To further optimize the programme, the practitioners recommended redistributing some of the assessment tasks between them, and to do some examinations more thoroughly.

## Discussion

Overall, the programme turned out to be acceptable and feasible for both practitioners and participants. The results of our study show that the programme was largely performed according to protocol. The medical and occupational-therapy assessments led to an average of 3.85 recommendations and/or referrals per participant. However, the number of referrals and recommendations ensuing from the medical assessments was relatively small (on average 0.58) compared to the recommendations ensuing from the occupational-therapy assessments (on average 3.57). Participants' self-reported compliance with the advice to contact their GP to be informed of the recommendations and/or referrals from the medical assessment was low to moderate (53%). Participants who were informed by their GP of the referrals and recommendations reported reasonable to good compliance (75%) with these referrals and recommendations. Participants' self-reported compliance with the recommendations they received from the occupational therapists was moderate (59%). Participants' overall compliance with the recommendations and/or referrals ensuing the medical and occupational-therapy assessments was 60%. Both participants and practitioners judged the programme to be feasible. A large majority of participants reported that they had benefited from the programme.

This process evaluation has provided insight into process-related factors that may explain the lack of effectiveness of our programme. The main process-related factors that may be responsible for the lack of effectiveness are the relatively low numbers of referrals and recommendations ensuing from the medical assessments and participants' poor compliance with the suggestion to contact their GP to be informed of the recommendations and/or referrals resulting from the medical assessment.

The limited number of referrals and recommendations ensuing from the medical assessments may indicate that our study population possibly was relatively healthy and not at high risk for falls and/or already received sufficient medical care. The inclusion criteria of our study and the study of Close et al [16] were comparable, although we additionally excluded participants who were permanently bedridden, fully dependent on a wheelchair, and were not able to complete questionnaires or interviews by phone. Comparison of our population with the population of Close et al[16] revealed that the number of recurrent fallers in our control group was comparable to the control group of Close et al. and other studies [5,16,18,19]. It is therefore unlikely that differences in population are the only explanation for the limited number of recommendations. It is possible that also differences in regular care in both countries can explain the limited number of recommendations. Possibly regular care in the Netherlands at the time of the study (2002–2005) was better than the regular care in the UK at the time of the study (1995–1998).

There are various possible explanations for the participants' low compliance with contacting their GP. Participants reported that the most important reasons for not contacting their GP were forgetting to do so, not thinking it useful, and not being aware of the possibility. These reasons may be related to the relatively long period between randomization and the moment the GPs were informed of the results of the assessments (on average 3.5 months). Recommending the participants to contact their GP and sending a subsequent reminder to all participants was apparently not sufficient to stimulate the participants to contact their GP. For our programme, this implies that it is not efficient to let the GPs act as intermediaries between the practitioners doing the assessments and the participants. However, our reason for incorporating the GPs was that we wanted to make the programme fit in easily with regular healthcare. In the Netherlands, referrals to medical specialists are implemented through a patient's GP [20]. In addition, GPs are familiar with the health status of their patients and can therefore act as supervisors to provide the best possible care. With hindsight, including GPs in the procedure seems to be an inefficient option, and is likely to have contributed to the lack of effectiveness of our trial. In the British version of the programme, Close et al. referred their patients directly to other services or a day hospital for further investigation, assessment or follow-up [16]. In the UK, as in the Netherlands, rehabilitation services include examinations, treatment and counseling by medical specialists, paramedical staff and behavioural or rehabilitation therapists. The major advantage of the British day hospital approach is that it produces "a one-stop shop" for patients with complex needs, which would otherwise (like in the Netherlands) require multiple visits to different departments, or multiple visits to GP's, medical specialist and therapists [21].

The present study had some possible limitations. First, participants and practitioners may have given socially desirable answers. We tried to avoid this tendency among participants by gathering data anonymously and by informing them that their answers would not affect their future use of healthcare services. Among practitioners, we tried to avoid social desirable answering by stressing that their comments and recommendations would only be used to improve the programme and not to judge their professionalism. A second limitation of this study is that we did not collect data directly from the GPs. We may have missed relevant data concerning the role of the GPs in the programme, e.g. whether the GP agreed with the suggested referrals and recommendations, and whether the participants actually called them.

## Conclusion

Based on the results of this process evaluation and the lack of effectiveness of our programme we do not recommend implementing the programme in its present form in regular care. We recommend two major adjustments to the programme. Firstly, we recommend to screen the potential participants of the programme on their fall risk by a routinely performed short fall risk screening among patients who attend the A&E department because of a fall [22-27]. Hence it should be possible to discriminate between a low to moderate risk group and a high risk group among community dwelling fallers who attending the A&E department. Focusing on fallers with a substantially increased risk of recurrent falls may improve the efficiency of the programme. Secondly, we aim to increase the efficiency of the programme by drastically decreasing the time between the patient attending the A&E department and the implementation of the fall prevention measures. We therefore recommend to perform the medical assessment preferably within two weeks after attending the A&E department for those directly discharged home, and around discharge for those admitted to hospital after the fall. Furthermore, the occupational-therapy assessment should be performed preferably within two weeks after the patient is being discharged home. To further increase the efficiency, the geriatrician who performs the medical assessment should be permitted to refer patients directly to relevant services in stead of having the GP implement the referrals. The geriatrician and occupational therapist should send the GP a comprehensive report on the outcomes of the assessments and the actions already taken. This would allow the GPs to continue and coordinate the fall prevention measures initiated or implemented by the geriatrician and occupational therapist. A follow-up consultation with the geriatrician and occupational therapist after 6 months is recommended to assess the patient's current risk profile, to increase long-term compliance with fall prevention measures, and to take additional fall prevention measures if necessary. However, whether the recommended adaptations to the programme will be realizable and feasible in Dutch healthcare should be thoroughly explored, because the proposed procedure deviates considerably from usual procedures in the Netherlands. We therefore strongly recommend that both the feasibility and (cost-) effectiveness of this adjusted programme should be studied before implementing it in Dutch regular care.

## Competing interests

The authors declare that they have no competing interests.

## Authors' contributions

All authors contributed to the development of the design of this study. MB drafted the manuscript with input from the other authors. All authors read and approved the final manuscript.

## Pre-publication history

The pre-publication history for this paper can be accessed here:


